# Regulation of Cell Proliferation and Migration by miR-203 via GAS41/miR-10b Axis in Human Glioblastoma Cells

**DOI:** 10.1371/journal.pone.0159092

**Published:** 2016-07-28

**Authors:** Dhananjaya Pal, Debasmita Mukhopadhyay, M. Janaki Ramaiah, Pranjal Sarma, Utpal Bhadra, Manika Pal Bhadra

**Affiliations:** 1 Centre for Chemical Biology, CSIR-Indian Institute of Chemical Technology, Uppal Road, Hyderabad, 500007, India; 2 School of Chemical and Biotechnology, SASTRA University, Tirumalaisamudram, Thanjavur, 613401, India; 3 Functional Genomics and Gene silencing group, CSIR-Centre for Cellular and Molecular Biology, Uppal Road, Hyderabad, 500007, India; 4 Academy of Scientific and Innovative Research, Arunasafali Marg, New Delhi, 110025, India; University of Saarland Medical School, GERMANY

## Abstract

Glioma amplified sequence 41(GAS41) is a potent transcription factor that play a crucial role in cell proliferation and survival. In glioblastoma, the expression of GAS41 at both transcriptional and post transcriptional level needs to be tightly maintained in response to cellular signals. Micro RNAs (miRNA) are small non coding RNA that act as important regulators for modulating the expression of various target genes. Studies have shown that several miRNAs play role in the post-transcriptional regulation of GAS41. Here we identified GAS41 as a novel target for endogenous miR-203 and demonstrate an inverse correlation of miR-203 expression with GAS41 in glioma cell lines (HNGC2 and U87). Over expression of miR-203 negatively regulates GAS41 expression in U87 and HNGC2 cell lines. Moreover, miR-203 restrained miR-10b action by suppressing GAS41. GAS41 is essential for repressing p53 in tumor suppressor pathway during cell proliferation. Enforced expression of GAS41 produced contradictory effect on miR-203 but was able to enhance p53 tumor suppressor pathway associated protein. It was also found that miR-203 maintains the stability of p53 as knock down of p53 expression using siRNA resulted in down regulation of pri-miR and mature miR-203 expression. Conversely reconstitution of miR-203 expression induced apoptosis and inhibited migratory property of glioma cells. Taken together, we show that miR-203 is a key negative regulator of GAS41 and acts as tumor suppressor microRNA in glioma.

## Introduction

Gliomas are the most frequently occurring neuro epithelial brain cancer arising from glial cells in the brain. It accounts for 12–15% of all brain tumor [1–4] and are categorized into four grades (I–IV) according to World Health Organization (WHO) [5]. Among all glioma cases diagnosed, astrocytoma grade III or glioblastoma multiform (GBM) is considered to be the most severe and incurable form due to poor prognosis and high invasiveness. High levels of cellular heterogeneity due to genetic mutation or variation involved in the control of cell cycle, growth, apoptosis, invasion, and neo vascularization are also observed [6, 7]. Despite different strategies adopted for treatment, patients diagnosed with GBM are inevitable to result in the relapse of the disease [8–11]. Therefore further research in understanding the regulatory mechanism that disclose the molecular mechanisms of pathogenesis of glioblastoma is of utmost need.

MicroRNAs (miRNAs) are small non coding RNA that play a crucial role in the regulation of gene expression. They regulate gene expression by binding to the 3’ untranslated region of the cognet mRNA followed by translational inhibition [12–14]. Its role in association with tumorigenesis, angiogenesis, apoptosis and invasion for various types of cancer is well-established [15]. Misregulation of miRNA has been identified with numerous human cancers and deregulations of specific microRNAs have been associated with glioblastoma where they play dual role as oncogene and tumor suppressor [14]. miR-221, which targets tumor suppressor p27, is up regulated in GBM whereas miR-7 that targets epidermal growth factor (EGFR) is down regulated. Similarly, miR-124 and miR-137 are found to be down regulated but miR-21 is over expressed in GBM. Over expression of miR-10b facilitates invasive capability in high-grade glioma by suppressing HOXD10 and RhoC [16, 17]. miR-17-92 cluster that are frequently up regulated in glioblastoma show tumorigenic property by targeting anti proliferative gene, TGFBRII, SMAD4, and CAMTA1[18]. In addition, reduced expression of miR-7, miR-128 and miR-34c are linked to poor prognosis in glioblastoma multiforme. Some reports have also demonstrated down regulated expression of miR-203 is association with GBM.

miR-203 is known for its tumor suppressive activity by negatively regulating cell proliferation and invasion and enhancing chemotherapeutic intervention [19–22]. Recent studies have shown that down regulation of miR-203 is associated with chemo resistance in human glioblastoma by inducing EMT via SNAIL1. Over expression of miR-203 drastically suppress Robo1 which in turn suppress ERK phosphorylation and MMP-9 expression thereby repressing glioma cell invasion and migration by disrupting the Robo1/ERK/MMP-9 signaling cascade [20]. These clearly indicate that miR-203 plays a major role for maintaining glioma tumor cell migration and invasion putting up the probability of miR-203 to be a novel candidate for therapeutic development for gliomas.

Glioma Amplified Sequence 41 (GAS41), initially isolated from the glioblastoma multiforme (GBM) cell line is frequently amplified in glial tumors and is responsible for nearly 40% of tumor formation associated with central nervous system [23, 24]. It is found to be amplified in 23% of glioblastoma and 80% in grade I astrocytoma. GAS41 is highly conserved among species including humans, mice, *Caenorhabditis elegans* and *Drosophila melanogaster*. The protein contains an N-terminal tf2f domain that belongs to YEATS family member [23, 25]. GAS41is required for cell growth and survival and contributes to tumor progression by negatively modulating p53 pathway in normal cell cycle. It is essential for suppression of p53 during normal cellular proliferation by associating to the promoter of p14^ARF^and p21 [26]. Moreover GAS41 is an effective transcriptional factor that interacts specifically with the nuclear mitotic apparatus (NuMA) protein in the interphase nuclei and maintains the nuclear architecture. It possesses a transcription activation domain at its C terminal 90 amino acid but lacks DNA binding domain [27, 28]. GAS41 is also identified as a common subunit of TIP60 and SCARP7 complex and play a crucial role in cell survival and growth [29].

microRNAs are involved in several cellular processes and their misexpression is often seen in various tumors. Several studies have reported misregulation of miRNA in glioblastoma, but no relation between miRNA and GAS41 in glioblastoma been reported till date. In this report, we show GAS41 as a novel target for miR-203 where an inverse correlation between both occurs at transcription as well as translation level. Over-expression of miR-203 resulted in down regulation of GAS41 that lead to induction of p53/p21/p14 tumor suppressor pathway. Interestingly, miR-203 also down regulate miR-10b expression by repressing GAS41 in gliomas producing a proficient induction of apoptosis and preventing migration. All together this study put forward a new role of miR-203 as tumor suppressor miRNA that in turn control GAS41 expression in human glioblastoma cell line.

## Materials and Methods

### Cell culture

Human glioblastoma cell line (U87), normal human brain cell line (HCN2) were procured from American Type culture collection centre (ATCC, USA). HNGC2 glioblastoma stem cell line was developed at the National Centre for Cell Science (NCCS) Pune, India [30]. All cell lines were cultured in Dulbecco’s Modified Eagle’s medium (DMEM) (Sigma) supplemented with 10% fetal bovine serum (Life technology) and 100 mg / ml Penicillin and 100 mg/ ml Streptomycin sulfate (Sigma) and maintained at 37°C in a humidified chamber containing 5% CO_2_.

### Plasmid construct

The human precursor miR-203 sequence was obtained from *Ensemble* and amplified by polymerase chain reaction (PCR) using specific primers (S1 Table). The above PCR amplified fragment was cloned into EcoR1 and Xho1 restriction site of PLVXL-C1 lentiviral vector (Clontech). 3’ UTR of GAS41 was amplified from the genomic DNA using primer sequence as mentioned in S1 Table. The resulting PCR fragment was cloned into Xho1 and Not1 site of the psiCHECK-2 Vector. All the clones were confirmed by sequencing using gene specific primers. Mutant form of miR-203 binding site on GAS41 3’UTR was produced by base substitution in the primer of the miR-203 binding site followed by amplification and DpnI mediated site-directed mutagenesis [31]. Sequencing of the plasmid was performed for confirmation of the desired mutation. p21 full length promoter sequence was cloned into PGL3 basic vector (Promega) using kpn1 and HindIII restriction site for further studies. Two regions of p53 RES (-2500 to -1400) and (-1400 to +100) respect to TSS were cloned into kpn1 and Hind III site of PGL3 basic vector. Clones were confirmed by sequencing.

### Over expression of GAS41

Complete complementary DNAs of GAS41 gene was cloned into mammalian expression plasmid pCMV-Tag1 (Gift of Dr. Ramesh Ummani, CSIR-IICT, India). The ORF was PCR amplified by using the GAS41 specific forward and reverse primer. The PCR fragment was cloned into Not1 and BamH1 site to form GAS41-FLAG fusion construct. The clone was confirmed by sequencing. The plasmid was subsequently transfected to the cells and probed with Anti-FLAG antibody (Sigma) for to get the desired fusion protein.

### Luciferase assay

Cells were grown to 70% confluence and co-transfected with miR-203 construct and psiCHECK-2-GAS41 3’ UTR using Lipofectamine-2000 transfection reagent (life Technologies). After 6 h media was changed and cells were allowed to grow in normal DMEM media (10% serum containing). Luciferase assay was performed using dual luciferase reporter assay system (Promega) according to manufacturer’s instruction. Values were measured using Multimode Varioskan Flash instrument (Thermo-Fischer Scientific Ltd). Renilla luciferase values were normalized to firefly luciferase.

p21 promoter reporter assay was performed by incubating 2μg of p21 luciferase DNA construct and 0.5μg of β-galactosidase expressing vector with lipofectamine 2000 (Invitrogen) at room temperature in serum free DMEM for 6 hours after which the media was replaced with fresh DMEM containing 10% FBS. Cells were collected after 24 hours and lysed with RIPA buffer (Invitrogen) to measure the luciferase activity.

### Oligonucleotides transfection

24 h prior transfection U87 and HNGC2 cells were seeded at 2.5 × 10^5^ and 2.0 × 10^5^ cells per well respectively in 6-well plates and transiently transfected with GAS41siRNA, p53 siRNA and antimiR-203 at a final concentration of 50 nM using lipofectamine 2000 reagent (Invitrogen) according to the manufacturer’s instructions. One day after transfection, medium was replaced with fresh media containing 10% FBS and cells were incubated for 24h at 37°C.

### Western blot analysis

U87 and HNGC2 cells were transfected either with miR-203, GAS41 siRNA and FLAG-GAS41 overexpressed construct. Total cell lysates were obtained using ice-cold RIPA buffer (Sigma) containing protease inhibitor (Roche). After centrifugation at 12,000 rpm for 10 min, the protein in supernatant was quantified by Bradford method (BIO-RAD) using multimode Varioskan instrument (Thermo-Fischer Scientific). 40 μg of protein per lane was loaded in 12% SDS-polyacrylamide gel. After electrophoresis, the proteins were transferred to polyvinyldine difluoride (PVDF) membrane (GE Biosciences). The membranes were blocked at room temperature for 2 h in TBS + 0.1% Tween-20 (TBST) containing 5% blocking powder (Santa-Cruz Biotechnology) followed by washes with TBST for 15 min. Hybridization with primary antibody at recommended dilution was conducted; anti GAS41(1:1000), and anti β-actin (1:500) (purchased from Abcam and Novus bio), anti rabbit FLAG (1:1000) (purchased from Sigma), anti p53 (1:500), anti p53 ser 15 (1:1000), anti p21 (1:1000), anti p14 (1:1000), anti Bax (1:1000), anti Bcl_2_ (1:1000), anti cytochrome C (1:1000) (purchased from Abbiotech), anti E-cadherin (1:1000), anti MMP-9 (1:1000), anti Snail1 (1:1000),anti p53(1:1000) and anti caspase-3 (1:1000) (purchased from Cell Signaling Technology), Mdm2 (1:1000) (purchased from Origene), N-cadherin (1:1000) (purchased from Millipore) and incubated overnight at 4°C following which membranes were incubated with corresponding horseradish peroxides (HRP) labeled secondary antibody at room temperature for 1 h in dark. Blots were visualized with chemiluminescence reagent (Millipore) and observed by ChemiDoc XRS system (BIO-RAD)

### Immunofluorescence

HNGC2 and U87 cells were seeded on cover slips and allowed to grow for 24hours at 37^ο^C followed by transfection with miR-203 plasmid construct. Thereafter the cover slips were fixed with a para-formaldehyde solution (4% in 1X PBS) for 20 min at room temperature. Cell permeabilization was achieved by administration of Triton X-100 solution (0.2% in 1X PBS) for 5 min. Subsequently, cells were blocked with a 1% BSA solution for 60 min followed by incubation with anti-GAS41 antibody (1:200) at room temperature for 2 h. The cover slips were washed three times each for 5 min with PBST and incubated with FITC-conjugated anti-rabbit secondary antibody (Jackson Immuno Research Laboratories Inc) for 1 h. Thrice subsequent washes with PBST solution were performed. The cover slips were mounted on to slides with DAPI solution for nuclear staining. Cells were observed under confocal microscope (Olympus FV1000) and images were taken with the support of the flow view version 1.7c software program.

### Immunoprecipitation

Cells were grown in DMEM supplemented with 10% FBS. U87 and HNGC2 cell were transfected with EV and miR-203 construct by using lipofectamine 2000 (Invitrogen) in serum free media. Cells were lysed with RIPA buffer(sigma) and the cell lysate were immunoprecipitated with anti p53 antibody in conjunction to protein A sepharosebeads(GE).The beads were washed three times with lysis buffer and eluted with 5x SDS loading buffer. The immunoprecipitated product was subjected to western blot analysis.

### MicroRNA Expression

Total RNA was isolated from HNGC2, U87 and normal human brain cells. Equal amount of DNase-treated RNA was Poly-A tailed using Mir-X^TM^ miRNA first strand synthesis kit (Clonetech) according to the manufacturer’s protocol. Quantitative RT-PCR reaction was set up using universal reverse primer and miR-203 specific forward primer. The temperature conditions were 95°C for 10 min, followed by 40 cycles of amplification (95°C-15sec, 60°C-1 min).The relative expression level was calculated using ΔΔ Ct method in q-PCR (Applied Biosystem, 7900HT). The average of three independent analyses for each sample was calculated.

### Real Time PCR analysis

Total RNA was isolated by TRIzol® (life Technologies) method. RNA concentration and purity were measured by Nanodrop (Thermo Fisher Scientific Ltd). RNA clean up and DNase treatment was performed to get DNA free pure RNA. cDNA was synthesized using Superscript III first strand cDNA synthesis kit (life Technologies). Real-time PCR analysis was carried out using Applied Biosystem Power SYBR® Green PCR Master Mix in 7900HT Fast Real-Time PCR System (Applied Biosystem). Reaction mixture (final reaction volume 10 μl) was prepared using following components: 2 μl PCR-H_2_O, 1 μl forward primer (10 pmol), 1 μl reverse primer (10 pmol), 5 μl Power SYBR® Green PCR Master Mix and 1 μl template cDNA (50 ng). Amplification and quantification were carried out; Initial denaturation (95 °C for 1 min), amplification and quantification program repeated 40 cycles (95 °C for 10 s, 58 °C for 10 s, 72 °C for 20 s with each cycle fluorescence measurement mode), melting curve program (55 °C–95 °C) and finally cooling to 4 °C.

### Cell proliferation Assay

HNGC2 and U87 cells (5x10^3^ cells) were seeded in triplicate in 96 well plates in 100μl of DMEM medium for 24 h. Cells were transfected with miR-203 plasmid construct and empty vector and incubated for 24hr in a humidified incubator at 37^°^C. Cell proliferation status was measure by MTT assay kit (Life Technologies) on Varioskan flash multimode reader (Thermo scientific).The assay was measured in a time gap of 12hr, 24hr, 36hr and 48hr after transfection. The total procedure was repeated thrice and the average value was noted.

### Cell Cycle Analysis

5X 10^5^ HNGC2 and U87 cells were seeded in 60 mm dish and allowed to grow for 24 h followed by transfection with miR-203 plasmid construct. Cells were harvested with Trypsin-EDTA, fixed with ice-cold 70% ethanol at 40^◦^C for 30 min, washed with PBS and incubated with 1mg/ml RNase A solution (Sigma) at 37°C for 30 min. Further cells were collected by centrifugation at 2000 rpm for 5 min and stained with 250 μL of PI solution (Propidium Iodide) (10 mg of PI, 0.1 mg of trisodium citrate, and 0.03 mL of Triton X-100 at room temperature for 30 min in dark. The DNA contents of 10,000 events were measured by flow cytometer (Dakocytomation, Beckman Coulter). Histograms were analyzed using summit software.

### Measurement of Caspase-3 activities

Cells were seeded in 60mm dish and grown till they reached 60–70% confluence. Thereafter they were transfected with miR-203 plasmid construct. After 24hr of transfection, cell pellet was collected and washed twice with phosphate-buffer saline and suspended in RIPA lysis buffer (Sigma) containing protease inhibitor (Roche) for 15 min on ice. Subsequently cells were centrifuged for 15 min at 12,000 g in cold conditions. Supernatant was added to assay buffer (20 mM HEPES -pH 7.4, 2 mM EDTA, 0.1% CHAPS and 5 mM DTT) containing 40 μM of caspase-3 substrate, Ac-DEVD-AMC. The duration of incubation was for 1 h. Readings were recorded at an interval of 5 min. Fluorescence released by AMC was measured at 360 nm excitation spectra and 460 nm emission spectra respectively. Values were normalized to protein concentration and expressed as a percentage of activity relative to control. Values from three independent experiments are taken and shown as the mean ± standard deviations.

### Wound-heal assay

Both HNGC2 and U87 cells were seeded in 6-well plates and incubated for 24 h to reach confluence up to 85%. After 24 h a horizontal line in the centre of the well was scratched using a sterile plastic 200 μl micropipette tip.miR-203 plasmid construct was added at a final concentration of 50nM. The cells were incubated in growth medium several times at intervals of 24 h. At each time point image of the wound area were captured using an inverted phase-contrast microscope 2.11 [32].

### TUNEL assay

Apoptotic cells were detected by an in situ Apoptosis Detection Kit (Clonetech), based on terminal Deoxynucleotide transferase mediated dUTP nick-end labelling method (TUNEL). 1X10^5^cells were seeded in 6 well plates and incubated overnight at 37^°^C till they reach 70% confluence. Cells were transfected with miR-203 plasmid construct and incubated for 24 hour at 37^°^C. Subsequently cells were fixed in 4% par formaldehyde for 30 min, permeabilized in 0.1% Triton X-100, and labeled with fluorescein-12-dUTP using terminal deoxynucleotidyltransferase. The localized green fluorescence of apoptotic cells (fluorescein-12-dUTP) was detected under confocal microscope (Olympus, FV1000).

### Clonogenic assay

A minimum of 500 cells per well was seeded into 6 well plate. After 24 hr interval cells were transfected with miR-203 construct and the media was replaced with fresh media. Cells were allowed to grow for 10days in fresh media with regular changes at every alternate day. Subsequently cells were washed with 1x PBS and fixed with methanol. Fixed colonies were stained with crystal violate (0.5% Crystal Violet). Images were captured using ChemiDoc XRS system (BIO-RAD). Each experiment was conducted in triplicates [33].

### Statistical analysis

Statistical analysis was carried out using Microsoft excel to evaluate the significant difference between control and sample groups. All variables were analyzed as independent experiments. The results were reported as mean ± SD. * represents p-value < 0.05, ** represents p-value < 0.01, *** represents p-value < 0.001.

## Results

### Inverse correlation of GAS41 and miR-203 expressions in human glioblastoma cell lines

**Fig 1:**
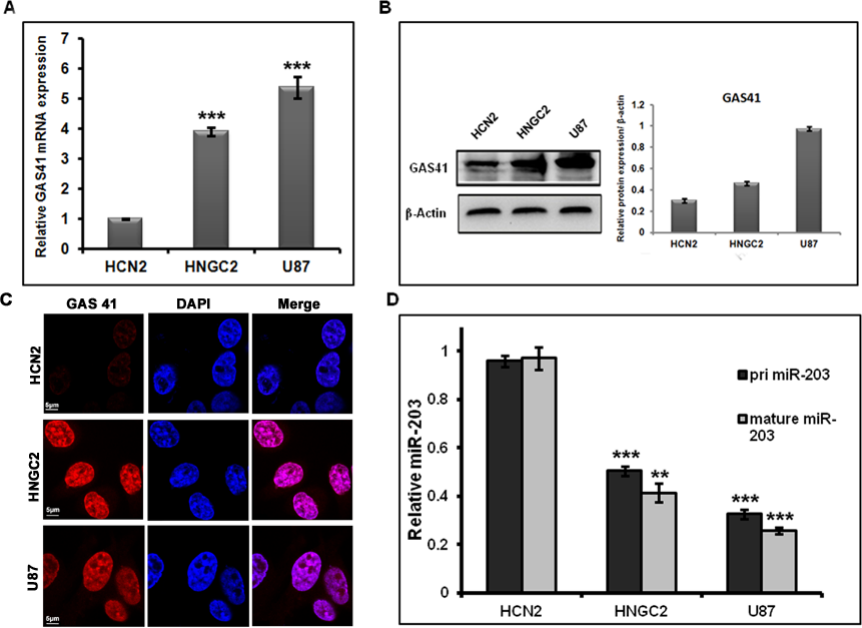
Inverse correlation between GAS41 and miR-203. **(A**) Real-time PCR analysis of GAS41 mRNA expression in HNGC2 and U87 glioblastoma cell line compared to normal cell. Bars represent relative GAS41 mRNA level normalized to GAPDH RNA from same sample. **(B)** Western blot analysis demonstrating GAS41 protein expression in HNGC2, U87 and normal HCN2 cell line. Bars represent mean average of relative GAS41 protein expression normalized to β-actin loading control from three independent experiments. **(C)** Immuno localization studies of GAS41 protein in HNGC2, U87 and normal cell lines. Scale bars represent 5μm. **(D)** RT-PCR studies showing the expression of pri-miR-203 and mature miR-203 in HNGC2, U87 and normal cell line. Bars represent relative pri-miR-203 and mature miR-203 level normalized to U6 RNA in the same sample. ** represents p value <0.01, *** represents p value <0.001 vs normal cell line. All experiment was conducted in triplicates.

Comparative gene expression study is done routinely to understand and characterize disease conditions especially in cancer. It is highly useful in identifying subtypes and developing strategies for better therapeutics.We initially examined the expression of GAS41 at transcriptional level by means of Quantitative RT-PCR in human glioblastoma cell line and corresponding non cancerous glial cell. We observed a distinct up regulation in GAS41 mRNA level both in HNGC2 and U87 cell lines compared to the normal cell **(Fig 1A).**Western blot analysis also showed similar up regulation of GAS41 in U87 and HNGC2 glioblastoma cell lines as compared to normal cell line demonstrating a clear retention of GAS41 expression at the translational level in both glioblastoma cell lines **(Fig 1B).** Further immunostaining experiments using GAS41 antibody also produced a similar pattern of expression in both HNGC2 and U87 cell lines (**Fig 1C**). We next measured the endogenous level of miR-203 in the same glioblastoma cell lines (HNGC2 and U87) by performing quantitative real time PCR (qRT-PCR). To demonstrate whether congregation of miR-203 is at transcriptional or post-transcriptional level, we analyzed the expression level of both primary and mature form of miR-203. There was a sharp depletion of primary and mature miR-203 expression in HNGC2 and U87 cells compared to normal human brain cells **(Fig 1D)** indicating an inverse correlation of miR- 203 with GAS41 in both HNGC2 and U87 cell lines.

### GAS41 is a novel target of miR-203

**Fig 2:**
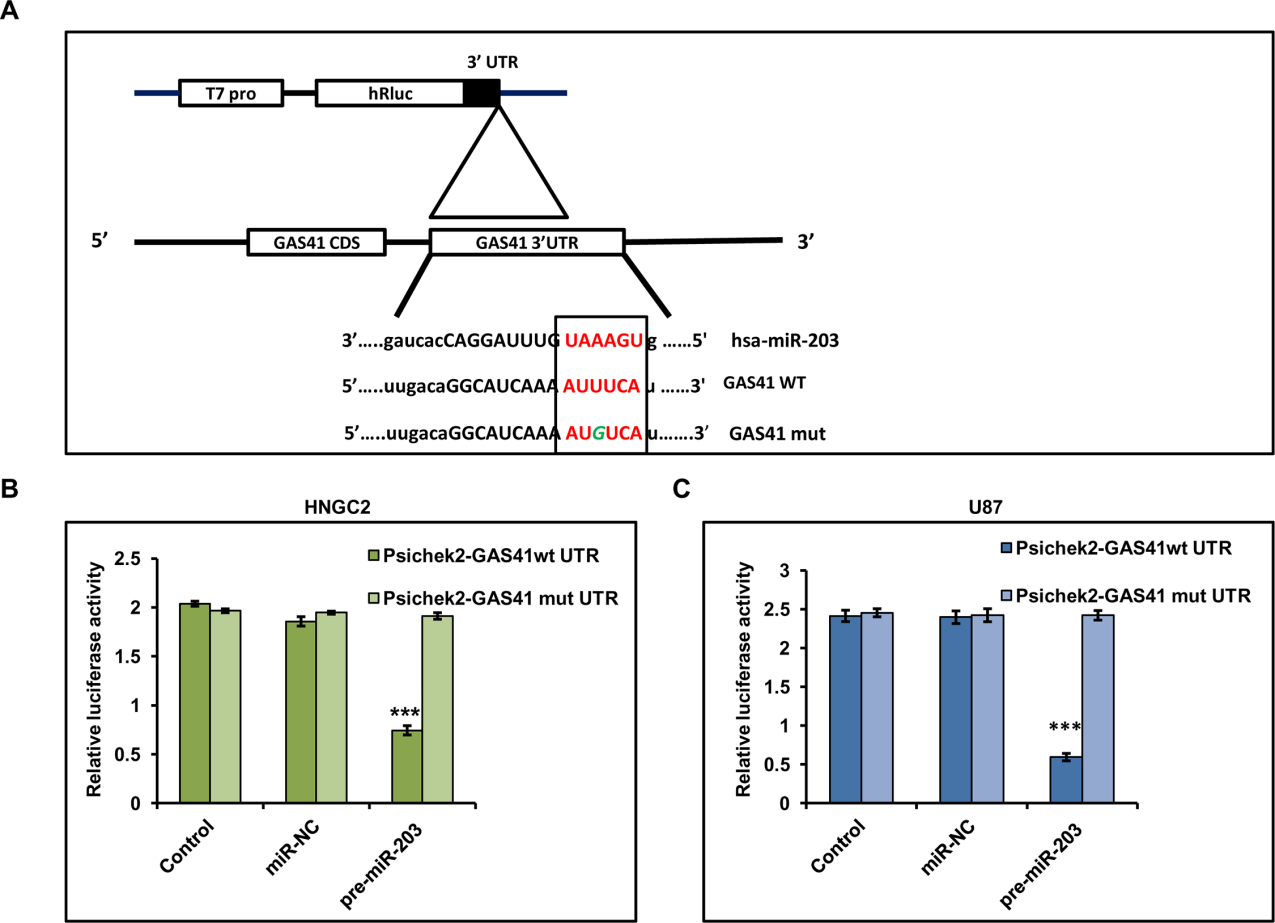
miR-203 mediated GAS41 Regulation. **(A)** Schematic representation depicting the cloning scheme of 3’-UTR of GAS41 (wild type and mutant) into luciferase ORF of psiCHECK2 vector. **(B & C)** Reporter Luciferase expression in HNGC2 and U87 cells co transfected with miR-203 and 3’UTR of wild type (WT) GAS41 compared to cells co transfected with miR-203 and 3’UTR of mutant GAS41 (MT). U87 and HNGC2 cells co transfected with blank vector or negative control miRNA (NC) and 3’-UTR of wild type or mutant GAS41 served as control. *** represents p value <0.001 vs. control.

GAS41 is frequently amplified in human glioblastoma and Grade 1 astrocytoma [23]. Fischer et. al. reported that over expression and amplification of GAS41 leads to glioblastoma recurrence[34]. miRNA expression is often deregulated in several glioblastoma. These changes in expression are often linked to the modulation of expression of oncogenes or tumor suppressor genes. In order to scrutinize whether expression of GAS41 is regulated by any known or unknown miRNA, we underwent in silico analysis using MiRanda (http://www.microrna.org) to identify the presence of any miRNA seed matching sequences within GAS41. We found only one putative target binding site of miR-203 (297–317) in the 3’UTR of GAS41 **(Fig 2A)**. This initial observation leads us to speculate that miR-203 may play a significant role in interfering with GAS41 expression in human glioblastomas. To address this we cloned 3’UTR region of GAS41 (GAS41-wt) into Promega psiCHECK2 luciferase vector and further co transfected to HNGC2 and U87 cells with miR-203 mimic. Result showed a significant depletion in luciferase activity as compared with control (cells transfected with empty psiCHECK2 vector). In contrast, HNGC2 or U87 cells co-transfected with miR-203 mimic and mutant 3’UTR of GAS41 construct (GAS41-mut) did not show any change in luciferase signal as compared to control confirming the connectivity of expression of GAS41 with mir203 expression **(Fig 2B & 2C).** This clearly delineated that miR-203 specifically binds to the 3’UTR of GAS41 in glioma cells which in turn establishes the association of mRNA—miRNA at post transcriptional level which could lead to suppression of GAS41.

### miR-203 negatively modulate GAS41 in glioblastoma cell line

**Fig 3:**
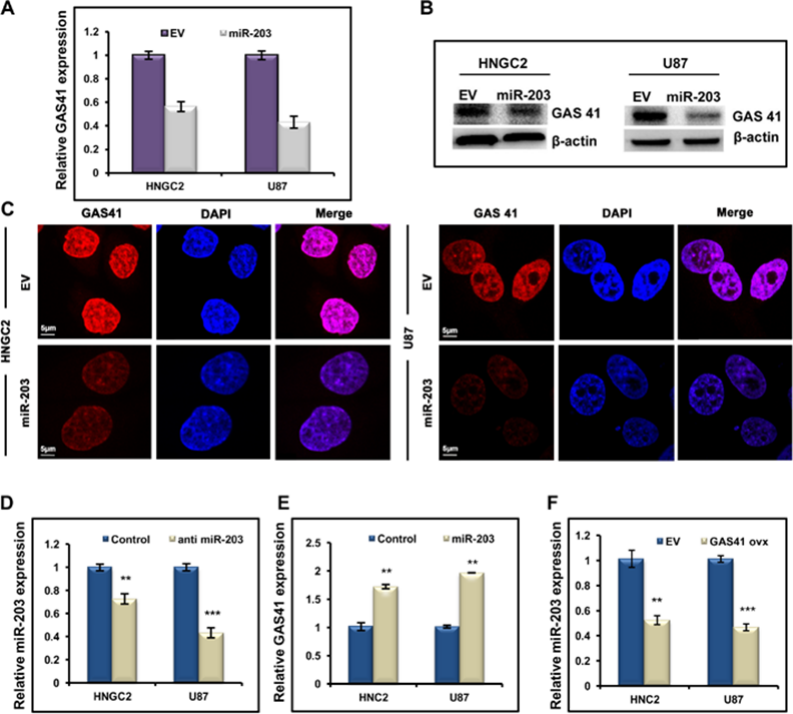
miR-203 down regulates GAS41 expression in glioblastoma cell. **(A)** Real time PCR studies showing the expression of GAS41 in HNGC2 and U87 cells transfected with miR-203. GAPDH is used as loading control in the experiment.Transfection of both the cell lines with empty vector (EV) served as control. Bars represent relative GAS41 mRNA expression normalized to GAPDH from same sample. ** represents p value<0.01 vs empty vector transfected control. **(B)** Western blots showing expression of GAS41 protein in HNGC2 and U87 cells transfected with miR-203 mimics. **(C)** Immunofluorescence studies showing the expression and localization of GAS41 in HNGC2 and U87 cells transfected with miR-203. Scale bars represent 5μm. **(D)** Quantitative RT-PCR studies showing expression of miR-203 in HNGC2 and U87 cells transfected with antimiR-203. *** represents p value <0.001, ** represents p value <0.01vs control. **(E)** Quantitative RT-PCR studies showing expression of GAS41 after exposure to anti-miR-203 in HNGC2 and U87 cells. ** represents p value **<**0.01vs control **(F)** Quantitative Real time PCR studies showing expression of miR-203 in HNGC2 and U87 cells upon over expression of GAS41.** represents p value <0.01 and *** represents p value <0.001 vs EV (cells transfected with pCMVTag1 vector). All the above experiments were performed in triplicates.

To further demonstrate that miR-203 negatively regulates endogenous GAS41 expression, we over-expressed miR-203 in both glioblastoma cell lines (HNGC2 and U87) and estimated its expression level by performing semi-quantitative RT PCR **(S1 Fig)**. We observed a sharp fall in GAS41 expression at transcriptional level compared to control (EV) **(Fig 3A).** Conversely, depletion of GAS41 protein level was observed when both the glioblastoma cell lines were subjected to miR-203 over expression **(Fig 3B**). In order to verify and visualize the effect we performed immunofluorescence staining studies in both the glioblastoma cell lines having over expressed GAS41 using anti GAS41 antibody **(Fig 3C).** Results clearly showed a reduced GAS41 signal upon miR-203 over expression, whereas no change was observed in glioblastoma cells transfected with empty vector. Expression of endogenous miR-203 is low in Glioma cells. Therefore it was our interest to see whether down regulation of miR-203 could revert GAS41 expression in Glioma cells. Thus we obscured miR-203 level by introducing antagomiR-203 in both HNGC2 and U87 cell lines **(Fig 3D).** Transfection with miR-203 in both the cell lines amplified GAS41 mRNA expression **(Fig 3E).** To facilitate GAS41- miR-203 regulationprototype, we over expressed GAS41 in glioblastoma cell lines by transfecting with pCMV-GAS41 construct which contains GAS41 coding region fused with C terminal FLAG into pCMV-Tag1 vector **(S1 Fig)** and evaluated miR-203 expression level. A clear decrease in the level of miR-203 expression was observed in cells transfected with over expressed GAS41 as compared to EV (pCMV Tag1) **(Fig 3F)**.These result suggest that miR-203 expression is indeed bowed down in glioma as GAS41 expression is predominant in these cell types.

### miR-203 down regulates miR-10b expression via GAS41

**Fig 4:**
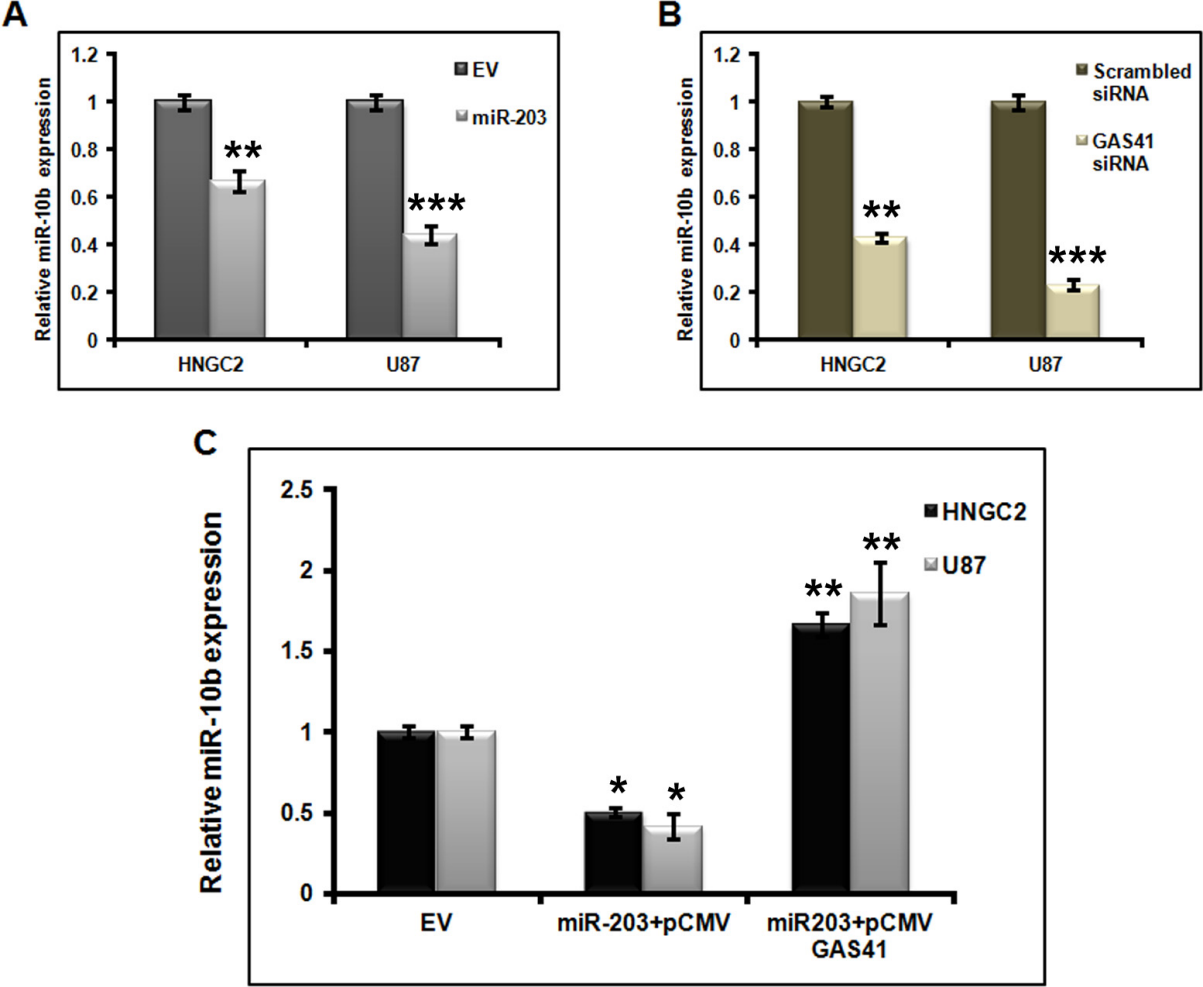
GAS41 mediates suppression of miR-10b through miR-203. **(A)** Quantitative Real time PCR analysis showing the expression of miR-10b in HNGC2 and U87 cells transfected with miR-203 plasmid. Bars represent relative miR-10b expression normalized toU6 snRNA. ** represents p value <0.01, *** represents p value <0.001 vs empty vector (EV). **(B)** Quantitative RT-PCR analysis showing the expression of miR-10b in HNGC2 and U87 cells transfected with scrambled siRNA and GAS41 siRNA (GAS41si). U6sn RNA is used for normalization. ** represents p value <0.01, *** represents p value <0.001vs scrambled siRNA. **(C)** qRT-PCR analysis showing expression of miR-10b in HNGC2 and U87 cells transfected with constructs carrying over expressed GAS41.* represents p value <0.05, ** represents p value <0.01 vs. EV (empty vector). All the above experiments were performed in triplicates

Evidences show altered microRNA 10b expression in breast cancer and GBM. However, the mechanistic properties of miR-10b in glioblastoma occurrence are not well understood [16, 35]. To demonstrate whether miR-203 has any connection in the modulation of miR-10b expression in glioma cell lines, we performed miR-10b expression studies in HNGC2 and U87 cell lines transfected with miR-203 and empty vector (EV) by performing quantitative real time PCR. We observed a clear depletion in miR-10b level in miR-203 transfected U87 and HNGC2 cells. **(Fig 4A)**, which suggest that miR-203 act as a potential negative regulator for miR-10b. As our initial observationshowed that GAS41 is directly targeted by miR-203, our next question was to see whether miR-203 also down regulated the expression of miR-10b by repressing GAS41. We therefore transfected both the cells with GAS41 siRNA, that specifically suppress endogenous GAS41 expression and performed real time PCR to see the expression of both GAS 41 and miR 10b. Same experiment was performed with cells transfected with scrambled siRNA that served as control. As expected, a significant down regulation of GAS41 mRNA expresssionwas detected in both glioblastoma cell lines compared to control **(S2 Fig).** Simultaneously there was a clear significant down regulation in the expression of miR -10b in the same cell lines compared to controls transfected with scrambled siRNA **(Fig 4B)**.

Next we sought to examine whether GAS41 restoration could positively retain miR-10b expression in both the glioblastoma cell lines. We observed a clear enhancement of miR-10b expression in cells co-transfected with miR-203 and GAS41 over expressing construct. Simultaneously no change was observed in cells transfected with miR-203 and pCMV-Tag1 empty vector **(Fig 4C).** This clearly explained a link of miR-10b in regulating the expression of GAS41. On the whole as miR-203 expression controls the expression of GAS41 which in turn controls the expression of miR 10b, it could be certain that miR 203 is a critical entity that regulates the expression of the oncogene GAS41 and oncogenic miR-10b in glioblastoma.

### miR-203 mediated GAS41 suppression promotes activation of p53 tumor suppressor pathway

**Fig 5:**
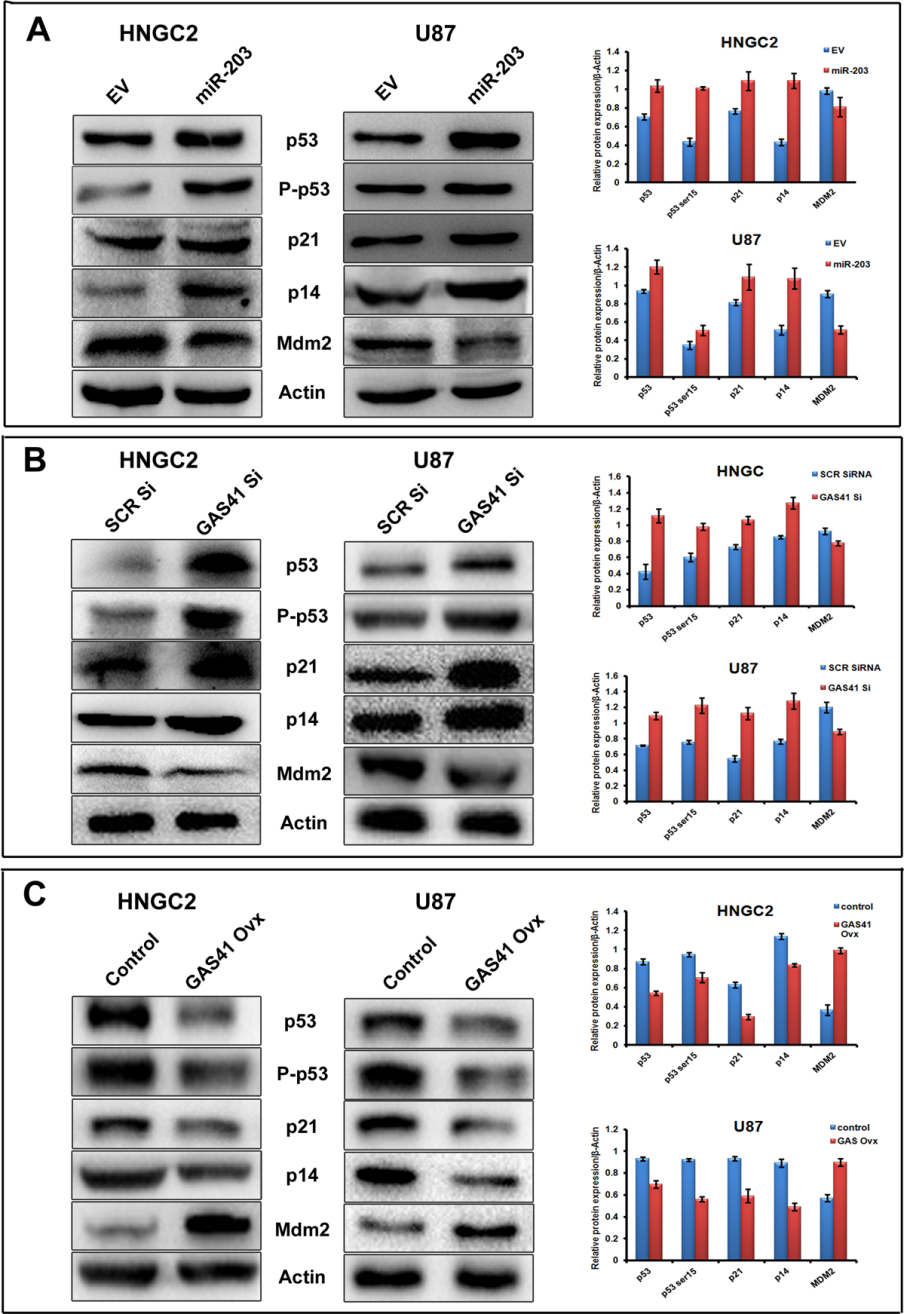
miR-203 induces p53 pathway by targeting GAS41. **(A)** Western blots analysis showing the expression of proteins related to p53pathway (p53, P-p53, p21, p14, Mdm2) in HNGC2 and U87 cells transfected with mir-203. Bars represent relative protein expression normalized to β-actin (housekeeping gene as gel loading control) from same sample with SD. **(B)** Western blots analysis showing the expression of proteins related to p53pathway (p53, P-p53, p21, p14, Mdm2) in HNGC2 and U87 cells transfected withscrambled siRNA and GAS41 siRNA (GAS41si). Bars represent relative protein expression normalized to β-actin (housekeeping gene as gel loading control) from same sample with SD. **(C)** Western blots analysis showing the expression of proteins related to p53pathway (p53, P-p53, p21, p14, Mdm2) in HNGC2 and U87 cells transfected with empty vector (EV) and upon over expression of GAS41 (GAS41 OVX). Bars represent relative protein expression normalized to β-actin (housekeeping gene as gel loading control) from same sample with SD.

GBM arises from astrocytes[36]and is frequently associated with altered expression of p53 or with mutational inactivation or loss of function of p53 [37]. GAS41 play a critical role in normal cell proliferation by binding to the promoter of p14 and p21 [26]that further promotes suppression of p53. But its dislocation leads to normal gene activation. To explore if miR-203 could up regulate p53 tumor suppressor pathway by inhibiting GAS41 in GBM, we introduced pre- miR-203 in both glioblastoma cell lines used in the study and assessed the expression of GAS41 protein **(S3 Fig)** as well as the proteins related to p53 pathway by performing western blot analysis using antibodies against GAS 41, p53, phospo-p53 (ser15), p14, p21 and Mdm2. We observed elevation of p53, phospo-p53 (ser15), p14, p21 expression when miR-203 was over expressed, whereas the expression of Mdm2 was reduced compared to control in both HNGC2 and U87 cell lines **(Fig 5A).** We also observed a similar pattern of expression of p53 pathway protein in both cell lines **(Fig 5B)** when GAS41 was knocked down using specific siRNA **(S3 Fig)**. Conversely, restoration of GAS41 by overexpressing CMV-Tag1-GAS41construct **(S3 Fig)** had opposite effect on p53 tumor suppressor pathway **(Fig 5C**). All together the observation suggested that loss of function of GAS41 either by miR-203 or by specific siRNA resulted in activation of p53 pathway and over expression of GAS41 reverted the entire pathway and retained Glioma growth.

### miR-203 maintains p53 stabilization and loss of p53 impairs miR-203 expression

**Fig 6:**
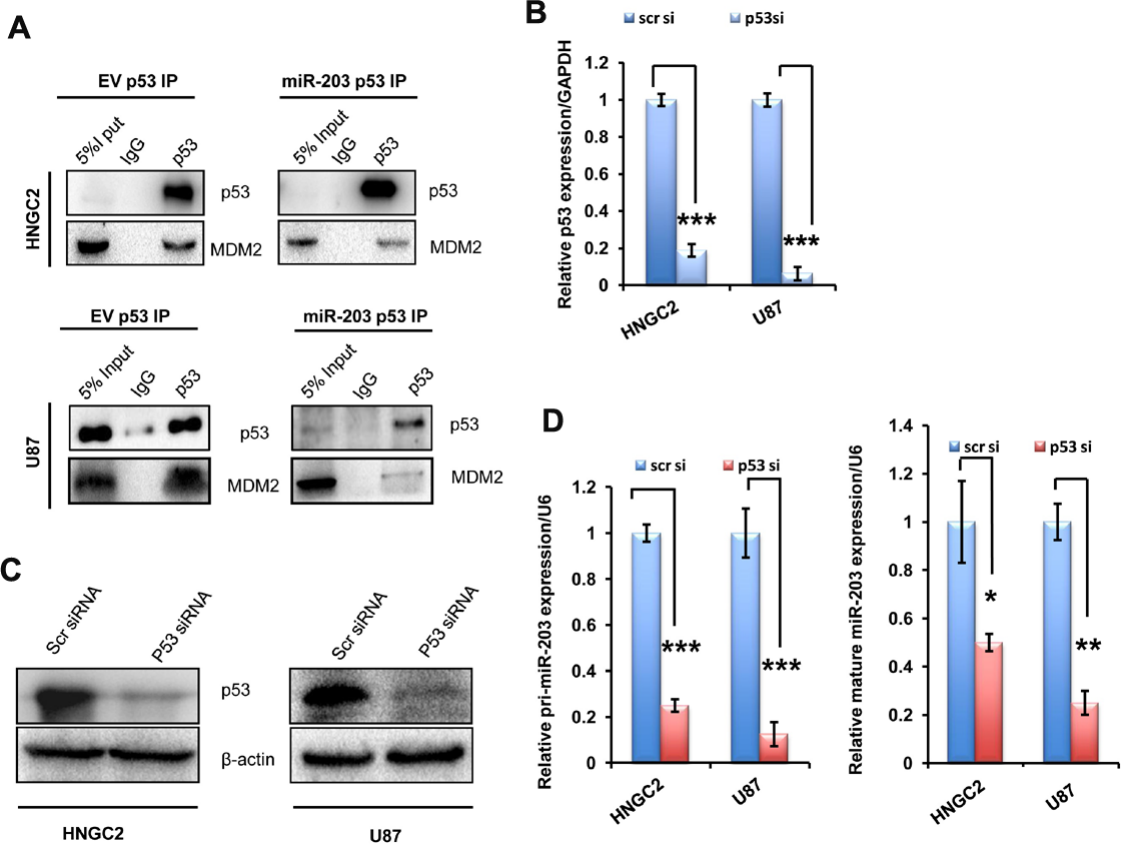
miR-203 dispensable for p53 stabilization. **(A)** Co immunoprecipitation studies using antip53 antibody in HNGC2 and U87 cells transfected with empty vector and miR-203 construct for 24 hr.The p53/MDM2 complex and the input was detected by westernblot. **(B)** Real time PCR analysis of p53 mRNA expression in HNGC2 and U87 cells transfected with p53 siRNA (p53si) and scrambled siRNA(scrsi) as control. GAPDH used for normalization. Bars represent RQ (2^-ddct^) of p53 expression normalized with GAPDH. ***represent the significant lable with p value <0.001 vs control.All experiments were conducted for at least three times. **(C)**Western blot analysis showing the level of p53 expression in HNGC2 and U87 cells transiently transfected with scrambled siRNA and p53 siRNA. β-actin **is** used as gel loading control **(D)** Quantitative PCR analysis of pri-miR-203 and mature miR-203 in cells introduced with scrambled siRNA (scrsi) and p53 siRNA (p53 si). Bars represent RQ (2^-ddct^) of pri-miR-203 and mature miR-203 expression normalized with U6. *** represents p value <0.001,**represent p value< 0.01,*represent p value <0.05. All the above experiments were performed in triplicates

Next we sought to examine whether miR-203 could stabilize p53 by interfering with intracellular p53/MDM2 interaction in U87 and HNGC2 cell line. We therefore performedCo-immunoprecipitation(Co-IP) studies where in p53 was immunoprecipitated from HNGC2 and U87 cells transfected with miR-203 and empty vector(EV) and immunoprecipitated p53 was hybridized with specific antibody against MDM2. A clear reduction in the expression of MDM2 level was observed in p53 immunoprecipitatesfrom cells exposed to miR-203 compared to control cells transfected with empty vectors **(Fig 6A),** demonstrating that miR-203 definitely interferes with the interaction of p53/MDM2, and there by promoting the degradation of MDM2 and leading to the stabilization and accumulation of p53. Our next vision was to sought whether expression of miR-203was dependent upon p53 or not. As both the cell lines used in the study contain wild type p53, we knocked down p53 expression by transfecting both the cell lines with p53 siRNA and checked the expression of p53 mRNA and protein in both the cell lines. A clear depletion of p53 expression at both mRNA and protein level was observed **(Fig 6B & 6C).**We simultaneously checked the expression of miR-203 in the same cells in which p53 expression was depleted by transfecting with p53 siRNA, A significant reduction in both primary and mature miRNA level of miR-203 was observed compared cells transfected with scrambled siRNA that served as controls **(Fig 6D)** suggesting a clear indication that p53 is required for miR-203 mediated cellular activities.

### miR-203 mediated GAS41 suppression lead to activation of p21 transcription

**Fig 7:**
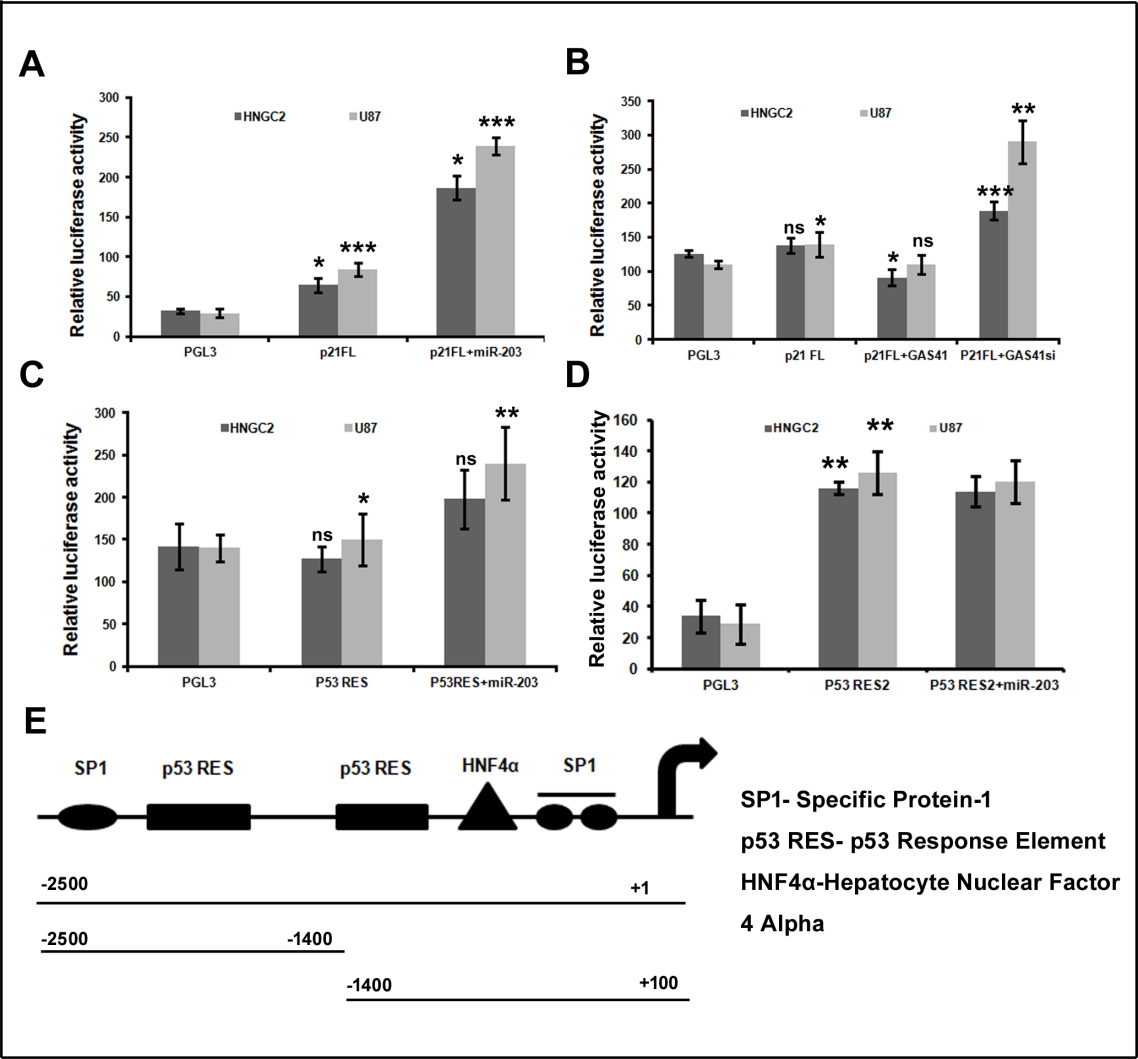
miR-203 regulate p21 promoter activity. **(A)** Luciferase assay showing p21FL promoter activity in HNGC2 and U87 cell line transfected with PGL3vector, p21FL promoter and p21FL (full length) plus miR-203. Bars represent relative luciferase activity normalized to β-gal.* represents p value <0.05, ** represents p value <0.01, *** represents p value <0.001 vs. PGL3 and p21FL. **(B)** Luciferase assay showing p21 promoter activity in HNGC2 and U87 cells transfected with miR-203 plus GAS41 and miR-203 plus GAS41 siRNA (GAS41si). Bars represent relative luciferase activity normalized to β-gal.* represent p value <0.05, ** represents p value <0.01,***p<0.001 vs. PGL3 and p21FL.ns represent non significant. **(C)** Luciferase assay showing p21 promoter activityin HNGC2 and U87 cells transiently transfected with -2500 to -1400 region containing p53 RES with miR-203. Bars determine relative luciferase activity normalized toβ-gal from three independent experiment.* represents p value <0.05,** represents p value <0.01. ns denotes non significant. **(D)** Luciferase assay showing p21 promoter activityin HNGC2 and U87 cells transiently transfected with p53RES2 (-1400 to +100) region along with miR-203. Bars represent relative luciferase activity normalized to β-gal from three separate experiments. ** represent p value <0.01 vs. PGL3 vector. **(E)** Schematic representation of p21FL (full length) promoter (spanning +1 to -2500kb) and two deletion construct from (-2500bp to -1400bp and -1400 to +100) cloned in PGL3 basic vector.

GAS41 mutation or its suppression by siRNA leads to activation of p21 and p14^ARF,^ indicating that GAS41 play a responsible role for repression of these genes. Although, the mechanism underlying the repression is not clearly known but some report state the involvement in promoter occupancy [26].So we wanted to know whether miR-203 could elevate p21 transcriptional level by inactivating GAS41, as p21 promoter shows a strong repression when associated with Gas41. We therefore co transfected full length p21 promoter and miR-203 mimic into HNGC2 and U87 cell lines. A clear increase in luciferase signal was seen when cells were co transfected with miR-203 mimics and p21promoter construct, whereas same cell lines introduced with empty vector and p21 promoter construct were unable to raise luciferase signal **(Fig 7A).** Surprisingly, when HNGC2 and U87 cells were co transfected with full length p21 promoter and GAS41expression construct, a marked reduction in luciferase signal was observed **(Fig 7B).** Similarly when HNGC2 and U87 cells were co transfected with p21 full length reporter construct and GAS41 specific siRNA, an increment of luciferase signal was similarly observed **(Fig 7B)**. These results confirmed that up regulation of p21 transcription mediated by miR-203 is indeed responsible for the down regulation of GAS41 by directly associating with it. Further, to determine whether p21 suppression by GAS41 is due to GAS41 occupancy in p53 response element at p21 promoter region, we developed two partial construct that contain p53 response element as p21 contains two p53 response elements in its promoter. We transiently co-transfected both the reporter construct in to HNGC2 and U87 cell line along with miR-203. We observed an increase in luciferase signal when cells were transfected with miR-203 and p53RES (-2500 to -1400) region **(Fig 7C),** whereas transfection with miR-203 and p53 RES2 (-1400 to +100) region was unable to respond to miR-203, producing no change in luciferase signal as compared to cells transfected with p53 RES2 alone **(Fig 7D)**. Hence p53RES (-2500 to -1400) is essential for activation of p21 promoter in response to miR-203.

### Over expression of miR-203 stimulates cell apoptosis and suppresses cell proliferation in human glioblastoma

**Fig 8:**
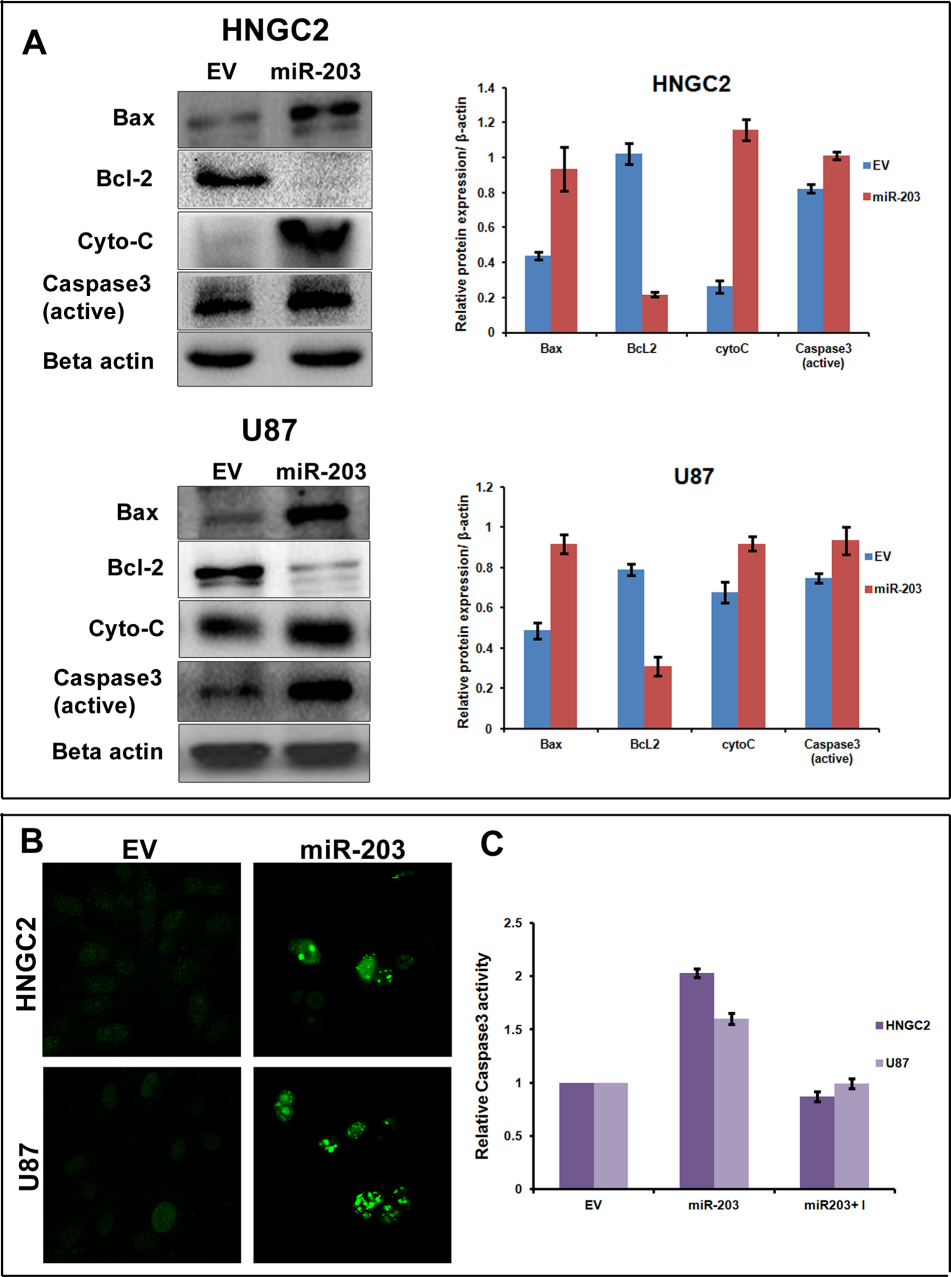
miR-203 induces apoptosis in glioblastoma cells. **(A)**Western blot analysis showing relative expression of proteins related to apoptosis (Bax, Bcl-2, Cyto-C, Caspase-3) in HNGC2 and U87 cells transfected with either empty vector or miR-203 over expressed plasmid.Beta actin served as housekeeping gel loading control. Bars represent the average of three independent experiments with SD. **(B)** Tunnel assay performed in HNGC2 and U87 cells transfected with miR-203 or empty vector showing a clear occurrence of apoptosis. **(C)** Caspase 3 activity in HNGC2 and U87 cells transfected with empty vector or miR-203 over expressing plasmid. Bars represent the average of three independent experiments with SD.

Previous reports have demonstrated that miR-203 act as a tumor suppressor miRNA and its expression is significantly down regulated in multiple cancers [38–41].It is also well documented that over expression of miR-203 could promote apoptosis and impair proliferation and migration [40, 41]. Though some report demonstrate silencing of YEATS4/GAS41 is capable of inducing apoptosis, but studies relating to association of miR-203 and GAS41 in glioma progression and apoptosis are still in its infancy. In order to see the possible role of miR-203 on cell apoptosis and cell proliferation in glioma cells, we performed cell viability assay after introducing miR-203 in both the glioma cell lines. Introduction of miR-203 resulted in decrease in cell viability as compared to empty vector (EV) transfected cell that served as control **(S4 Fig)**. Colony formation assay studies showed that upon transfecting both the cells with miR-203 plasmid a relatively less or no growth was clearly seen as compared to cells transfected with empty vector **(S4 Fig)**. We performed flow cytometry studies after transfecting both the cell lines with miR-203 along with untreated controls to measure the apoptotic cells. Five-fold increase of early apoptotic cells both in HNGC2 and U87 cell lines was observed when transfected with GAS41 as compared to EV transfected control **(S4 Fig).** To confirm the apoptotic event umpired by miR-203 over expression, we performed western blot analysis after isolating total protein from both the cell lines transfected with over expressed miR-203. Simultaneously western blots were also performed with proteins isolated from cells that were untransfected or transfected with empty vector that served as control. Blots were hybridized with antibodies against proteins that are responsible for inducing apoptosis. Results showed a clear up regulation of Bax, Caspase-3 and cytochrome C and down regulation of Bcl2 **(Fig 8A).** The observed result was further confirmed by performing TUNEL assay and caspase 3 assays which also showed a clear increase of apoptosis **(Fig 8B and 8C).**These results clearly showed that miR-203 play a imperative role in triggering apoptosis and combating cell growth and proliferation in glioma cell lines.

### Ectopic expression of miR-203 inhibits migration of glioma cells via GAS41

**Fig 9:**
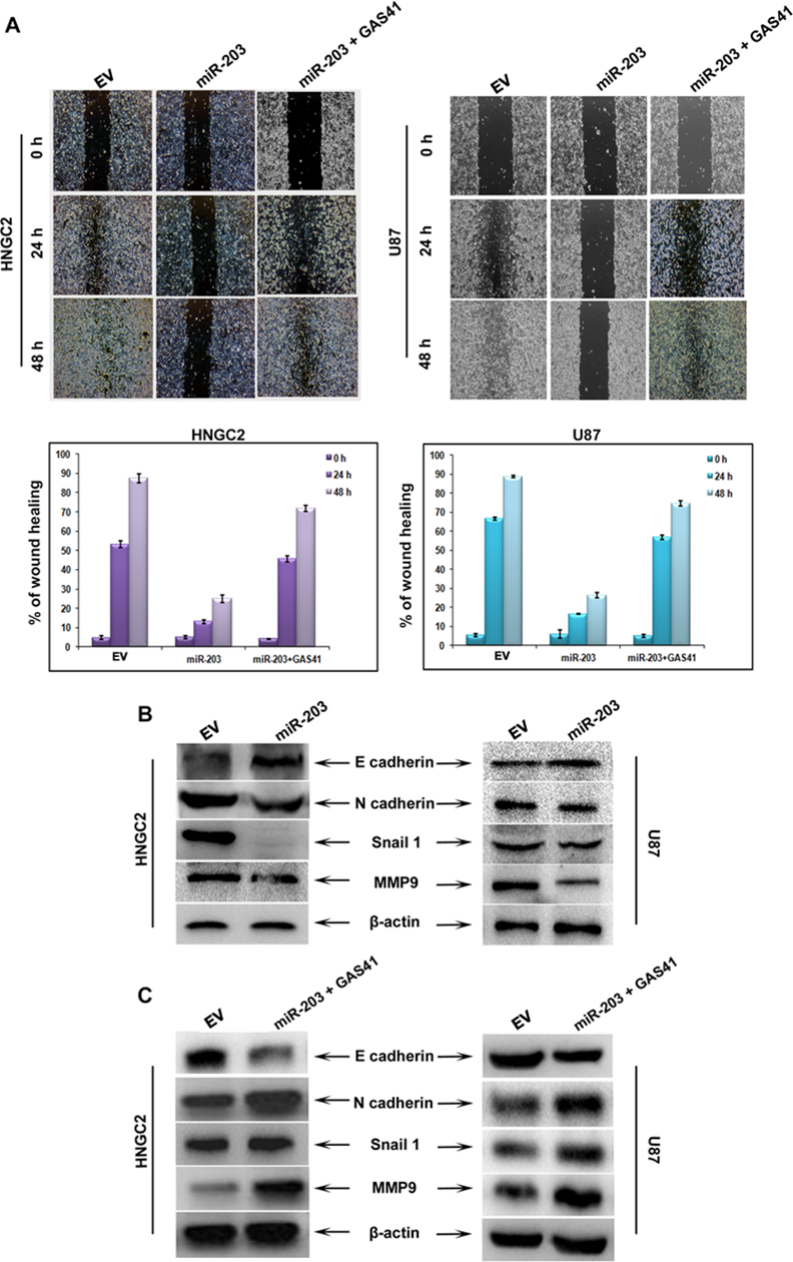
MiR-203 inhibit migration via GAS41. **(A)** Wound heal assay in HNGC2 and U87 cells transfected with empty vector (EV), miR-203 plasmid or miR-203 plus GAS41. Histograms representing the % of wound heal area in HNGC2 and U87 cells transfected with empty vector, miR-203 plasmid or miR-203 plus GAS41 **(B)** Western blots showing expression of proteins associated with migration event (E cadherin, N cadherin, Snail, MMP-9) in cells transfected with EV or miR-203. **(C)** Western blots showing expression of proteins associated with migration in cells transfected with either EV, miR-203 or miR-203 plus GAS41.

Migration and invasion are the key elements of brain tumor progression. In order to see the effect of GAS41 and miR-203 in tumor progression, we performed wound healing assay after reintroducing miR-203 mimic into both glioma cells. Glioma cells were transfected with empty vector control, miR-203 plasmid and miR-203 plus GAS41 construct and migration of cells were observed at specific time interval of 0h, 24h and 48h after transfection. Over expression of miR-203 virtually blocked the migration potential in HNGC2 and U87 cells as compared to empty vector transfected control **(Fig 9A)**, whereas cells transfected with GAS41 CDS resulted in aggressive migration. To reconfirm the result of migration assay and to see the effect at the translation level we performed western blot studies using antibodies against proteins that serve as markers for migration studies. Proteins were isolated from cells treated with miR-203 along with control samples and examined. There was an enhancement of E cadherin expression and reduction in the level of N-cadherin MMP 9 and snail1 upon miR-203 transfection in HNGC2 and U87 cell line **(Fig 9B).** Moreover, introduction of mi-203 plus GAS41 lead to restoration of migratory related protein marker **(Fig 9C).** These results confirmed that miR-203 attenuates glioma migration through inactivation of GAS41.

## Discussion

MicroRNAs play vital role in tumor progression and invasion. It regulates cognate gene expression by destabilizing target mRNA or producing translational inhibition through interaction with its 3’UTR. MicroRNAs are differentially expressed in cancers and they act variably either as oncogenes or tumor suppressor genes. Notably aberrant expression of miRNA is a central factor for couple of cancer type [14, 42]. Implementation of miRNA expression profiling and scrutinizing their function is a boon for tumor development and progression. Till date a very few miRNAs have been characterized for their specific role in cancer development, especially in glioblastoma.

miR-203 behaves as tumor suppressor and is down regulated in pancreatic, esophageal cancer and gliomas, whereas in epithelial ovarian cancer it is upregulated and act as oncogene. Here in our study we have reported the biological event of miR-203 in human glioblastoma. Our results demonstrate that miR-203 which is down regulated in glioblastoma cell lines combat apoptosis, and promote migration and proliferation potentially by targeting GAS41 *in vitro*. In support of earlier findings, our result also delineates that miR-203 function as a tumor suppressor in glioblastoma. The data depict Gas41 as a direct and functional target of miR-203which is commonly downregulated in glioma cell lines. We have also shown a unique role of GAS41 in connecting the functional relationship between miR-10b and miR-203. Our findings therefore suggest that upregulated expression of GAS41 along with concomitant reduction of miR-203 play a significant role in glioblastoma proliferation. Notably, restoration of miR-203 was able to up regulate of p53/p21 tumor suppressor axis, enhance apoptosis and inhibit migration through suppression of GAS41 probing a probability for better prognosis in glioblastoma.

Abnormal gene expression by allelic deletion on chromosome14 is a major cause of glioblastoma development. Earlier reports demonstrated over expression of miR-203 in prostate, laryngeal, and breast cancer cell lines [40, 41, 43]. It is also well established that miR-203 is down regulated in human glioma and restored expression of miR-203 could negatively regulate migration potential by targeting Robo1/ERK/MMP-9 Signaling cascade [20]. Nevertheless, miR-203 has also been implemented in esophageal and acute lymphoblastic leukemia cells that results in suppression of proliferative activity by targeting DNp63 and ABL1 [44, 45].Consistent with these reports, we have also found endogenous miR-203 is significantly down regulated in glioblastoma cell lines as compared to normal cell. miR-203 has several predicted protein targets that are oncogenic in nature. GAS41 is oncogenic in nature and highly amplified in human GBM. Its involvement is essential for glioblastoma growth. Moreover, GAS41 plays a pivotal role in RNAi pathway by interconnecting heterochromatin and miRNA biogenesis. Our data has shown a significant up regulation in GAS41 expression both at the mRNA and protein level, clearly reflecting an inverse correlation between miR-203 expression and GAS41 expression in glioma providing a possible clue for the over expression of this oncogene in human glioma.

To further understand and demonstrate the physiological relation between GAS41 and miR-203, and to understand the link of GAS41 to miR-203, we have used miRNA target prediction software to predict the target site of miR-203 with GAS41. This was further validated by assaying the luciferase activity of GAS41 wild type 3’ UTR expressing vector with a mutant miR-203. Mutant miR-203 showed no effect on GAS41 expression suggesting that miR-203 directly target 3’UTR of GAS41 and to suppresses its function.

GAS41 was initially discovered from the glioblastoma multiform cell line. It is highly amplified gene in human glioma [23]. GAS41 possess a C-terminal α-acidic activation domain, which is a hallmark of basic eukaryotic transcriptional factor but a lack of DNA binding domain. However the role of GAS41 in association with miRNA in the field of cancer remains unaddressed. To have an in depth understanding, we over-expressed miR-203 in glioma cells and analyzed GAS41 expression at both protein and RNA level. We observed a clear reduction in GAS41 expression. These results conclude that miR-203 negatively regulate GAS41 expression in human glioma.

A major reduction of miR-10b level was observed when miR-203 was over expressed in glioma cells. miR-10b was originally identified as a miRNA that is related to metastatic breast cancer. Presently its role as an onco miR in progression of other cancers is well explored [16, 35]. It is well established that in breast cancer, miR-10b suppress migration and invasion property by directly targeting HOXD10 and RHOC [35].It has been known that miR-10b also interacts with KLF4 to stimulate migration and invasion potential in esophageal squamous cell carcinoma[46]. Our present observation showed that over expression of miR-203 down-regulate miR-10b expression substantially. Simultaneously we found that GAS41 regulates miR-10b expression and miR-203 expression down regulated miR-10b expression by repressing GAS41. Knock down of GAS41 negatively cooperated with miR-10b expression and restoration of GAS41 resulted in miR-10b up regulation. Therefore we hypothesized that miR-203 expression inversely correlates with the expression of both miR-10b and GAS41 in glioblastoma cell (data not shown). However the molecular mechanism by which GAS41 regulates miR-10b expression need to be fully elucidated.

GAS41 and its homolog Yaf9 are integral part of the human TIP60 and yeast NuA4 complex. Loss of function or targeted disruption of this gene results in unusual effect in cell viability. Different subunits of NuA4 complex are required for yeast cell viability, whereas Yaf9 prefer to normal growth condition [25]. In comparison, GAS41 is crucial for cell growth and viability but misguidance of the gene has lead to cell death in chicken cell [27]. However knockdown by siRNA leads to growth arrest phenotype in HeLa cells, implying that, GAS41 is needed for cell growth and viability [47]. Moreover recent study demonstrated that GAS41 positively contribute in cell proliferation by binding to two p53 tumor suppressor pathway protein p21and p14. Here our results clearly depict that miR-203 mediated suppression of GAS41 effectively induced p53 expression as well as p21 and p14^*ARF*^gene. Knockdown of GAS41 by siRNA had the similar effect on p53 as well as p21 and p14^*ARF*^ expression. Whereas, opposite effect was observed upon over expression of GAS41 showing a clue for over amplified expression of GAS41 in glioblastoma in association with p53 tumor suppressor pathway.

p53 stabilization and activation depends on its association with and ubiquitination by MDM2 prior to proteosomal degradation[48].During cellular stress by exposure to genotoxic environment, p53 gets stabilized and undergoes post translational modification to be activated [49]. Once activated, it gets detached from MDM2, to make its route to the nucleus, where it interacts with the promoter of the target gene [50, 51]. Therefore avoiding ubiquitination of p53 by compromising its interaction with MDM2 is one of the important features for stabilizing p53.Our result also supported the notion that overexpression of miR-203 leads to reduction in p53/MDM2 interaction and p53 accumulation.

Various post translational modification regulate p53 function and are responsible for specific cellular outcome[52]. Recent study identified that upon exposure to DXR,p53 post-transcriptionally stimulate several miRNAs, such as miR-203,miR-16,miR-206,miR-103 by processing pri-miRNA to pre-miRNA mediated by Drosha in the nucleus[53]. Though it is well understood that p53 regulate miR-203 expression in various cancer cell linesvery little is known about its regulation in Glioma. Here our results show that down regulating the expression of p53 result in diminishing the expression of both pri and mature miR-203 in HNGC2 and U87 glioblastoma cell line. This further suggests that p53 restoration is dispensable for miR-203 mediated suppression of glioblastoma proliferation.

We have also assessed the transcriptional activity of p21 upon miR-203 over expression in glioblastoma cell line. Our results indicate that p21 transcriptional activation is probably due to miR-203 mediated suppression of GAS41, as over expression of GAS41 nullifies the transcriptional activation of p21. Furthermore our data suggests miR-203 mediated p21 activation is specific to p53RES (-2500 to -1400). All together our data strongly support miR-203 mediated p21 transcriptional activation by suppressing GAS41 though an in depth study for understanding the direct relation is needed.

Deregulation of miRNA associated with various cancers is a rapid development to the field of cancer therapeutics; thereby adding a new avenue to the understanding of the disease and its control. Here we have demonstrated that miR-203 serve as a tumor suppressor miRNA by negatively regulating GAS41. The function of miR-203 as a tumor suppressor miRNA has long been established in number of cancer cell lines [38–41]. It is also known that ectopic expression of miR-203 controls migration, proliferation and apoptosis in prostate cancer [38, 39]. In contrast, over expression of miR-203 leads to decreased cell viability and cell cycle arrest in G1phase in laryngeal cancer [40]. Moreover, expression of miR-203 suppressed cell proliferation and migration in triple negative breast cancer cell line [41]. However the role of miR-203 in inducing apoptosis in glioblastoma is enigmatic. Here our data showed that miR-203 over expression leads to apoptotic cell death in both U87 and HNGC2 glioblastoma cell line.

Gliomas are aggressive brain tumor in adult human central nervous system. Its ability to invade and migrate towards normal brain tissue makes the disease hard to treat. This is due to extreme production of number of protease and regulating ECM, creating communication to neighboring tissue and thereby allowing migration to other parts of the brain. Multiple inducers of EMT have been documented including TGF-β, Wnt/β-catenin, SNAIL1/SNAIL2, Twist and Talin [54]. Though role of miR-203 in case of cancer cell migration is broadly elucidated, but there is no report in context to GAS41 association. Here for the first time we have shown miR-203 proficiently target GAS41 and reduce cancer cell migration. Although it is evident that miR-203 is suppressed in prostate cancer [55] as well as colorectal carcinoma [56]**,** a recent report has also shown that loss of miR-203 leads to enhanced invasion and migration in breast cancer cell. More interestingly, evidences show that increased miR-203 expression is connected with poor survival in pancreatic tumor. In contrast, our study reveal that miR-203 suppress glioma cell migration by targeting GAS41.This study also explain the diversified function of miRNA function either as tumor suppressor or oncomiR. They might switch on or switch off depending upon their cellular and physiological contest or tumor type.

In conclusion the present study showed an interesting connectivity that miR-203 acts as a tumor suppressor in glioblastoma and its appearance is inversely correlated with GAS41 expression. Additionally our study suggests that GAS41 is a novel target of miR-203 which negatively regulates GAS41 expression in glioblastoma. We also postulated a linkage between miR-203, GAS41 and miR-10b an oncomiR abundantly expressed in multiple cancer including glioblastoma. In addition, we observed activation of p53 pathway gene upon GAS41 suppression by miR-203. It would be interesting to know how GAS41 is regulated by miR-10b or vice versa in cancer microenvironment.

## Supporting Information

S1 Fig miR-203 suppress GAS41. (A) Semi quantitative RT-PCR was performed to evaluate GAS41 expression. GAPDH used as loading control. (B) Western blots showing the Flag-GAS41 expression after cells were transfected with pCMV Tag1-GAS41 expression construct and.β-actin used as loading control.
(TIF)

S2 Fig GAS41 knockdown by siRNA. (A) qRT-PCR showing GAS41 mRNA expression in HNGC2 and U87 cell lines. Bars represent relative expression of GAS41 normalized to GAPDH. (B) Western blots showing GAS41 protein expression after cells were transfected with GAS41 specific siRNA.
(TIF)

S3 Fig GAS41 inhibition and overexpression. (A) Western blot analysis of GAS41 in HNGC2 and U87 cells transfected with Empty vector (EV) and miR-203 plasmid construct. (B) Western blot analysis showing the expression of GAS41 in HNGC2 and U87 after cells was introduced with scrambled siRNA (scr siRNA) and GAS41 siRNA (C) Western blot showing the over expression of GAS41 in HNGc2 and U87 after cells were transiently transfected with only vector and pCMV-Tag1-GAS41 over expressing construct. For all β-actin serve as loading control.
(TIF)

S4 Fig **miR-203 inhibit glioma proliferation and induce apoptosis** (A) Cell viability studies in HNGC2 and U87 cells after transfection either with empty vector or miR-203 over-expressing vector at different time intervals (12,24,48 and 72h) **(B)** Colony formation assay after miR-203 transfection into HNGC2 and U87 cells. (C) Flow cytometry analysis of cell cycle demonstrating apoptosis in HNGC2 and U87 cells after transfection with miR-203 or empty vector.
(TIF)

S1 Table Primer list. (DOCX)

## Abbreviations

miR-203: microRNA-203
GAS41: Glioma amplified sequence 41
miR-10b: microRNA 10b
GBM: Glioblastoma multiforme
qRT-PCR: quantitative real time polymerase chain reaction
UTR: untranslated region
siRNA: Small interfering RNA
p53RES: p53 response element sequence
