# Supplementary figures and images for: Regulation of Cell Proliferation and Migration by miR-203 via GAS41/miR-10b Axis in Human Glioblastoma Cells
Source: PLoS One. 2016 Jul 28;11(7):e0159092. doi: 10.1371/journal.pone.0159092 (PMC4965126; doi:10.1371/journal.pone.0159092)

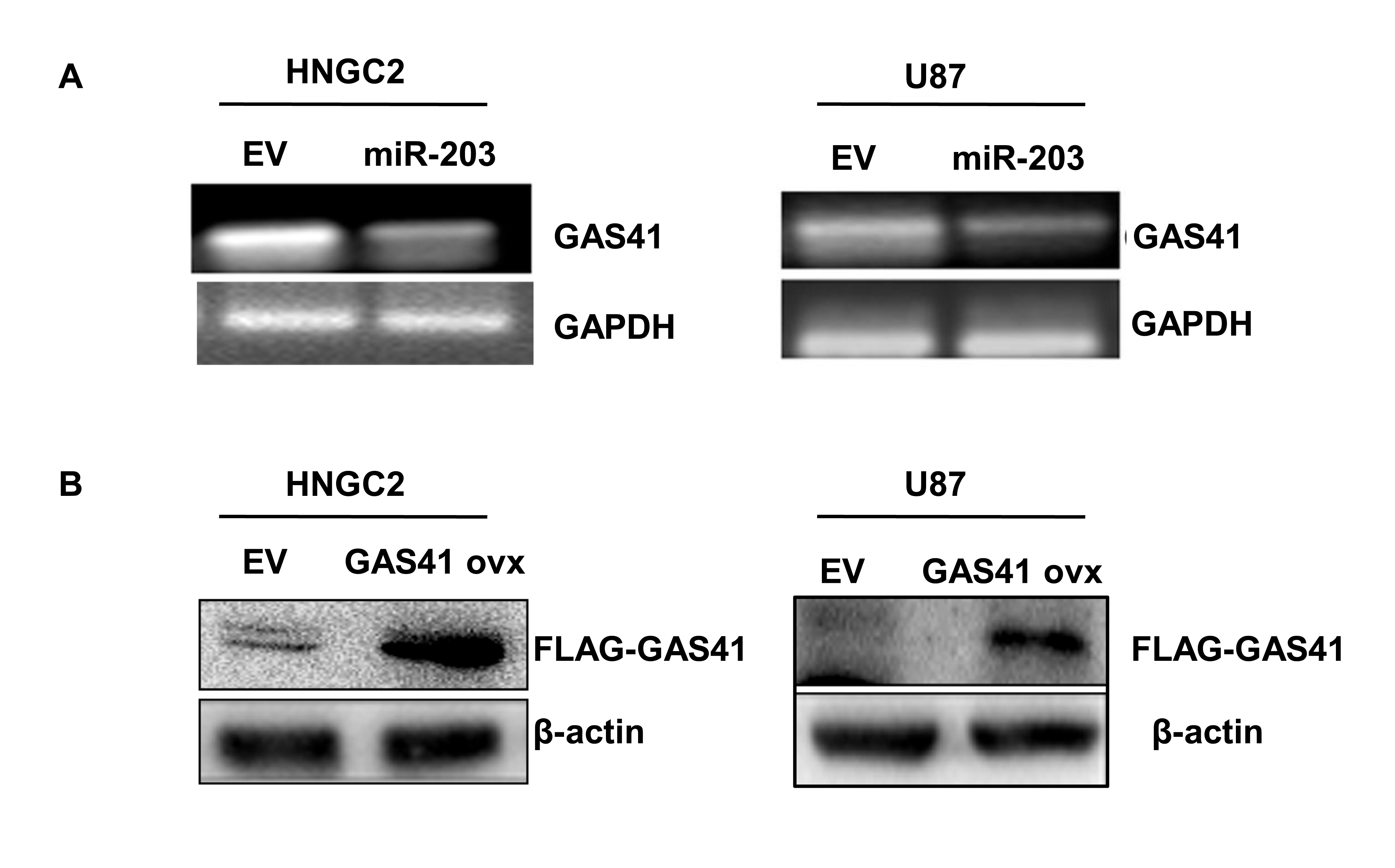

Supplement: S1 Fig — (A) Semi quantitative RT-PCR was performed to evaluate GAS41 expression. GAPDH used as loading control. (B) Western blots showing the Flag-GAS41 expression after cells were transfected with pCMV Tag1-GAS41 expression construct and.β-actin used as loading control. (TIF) [file pone.0159092.s001.tif]

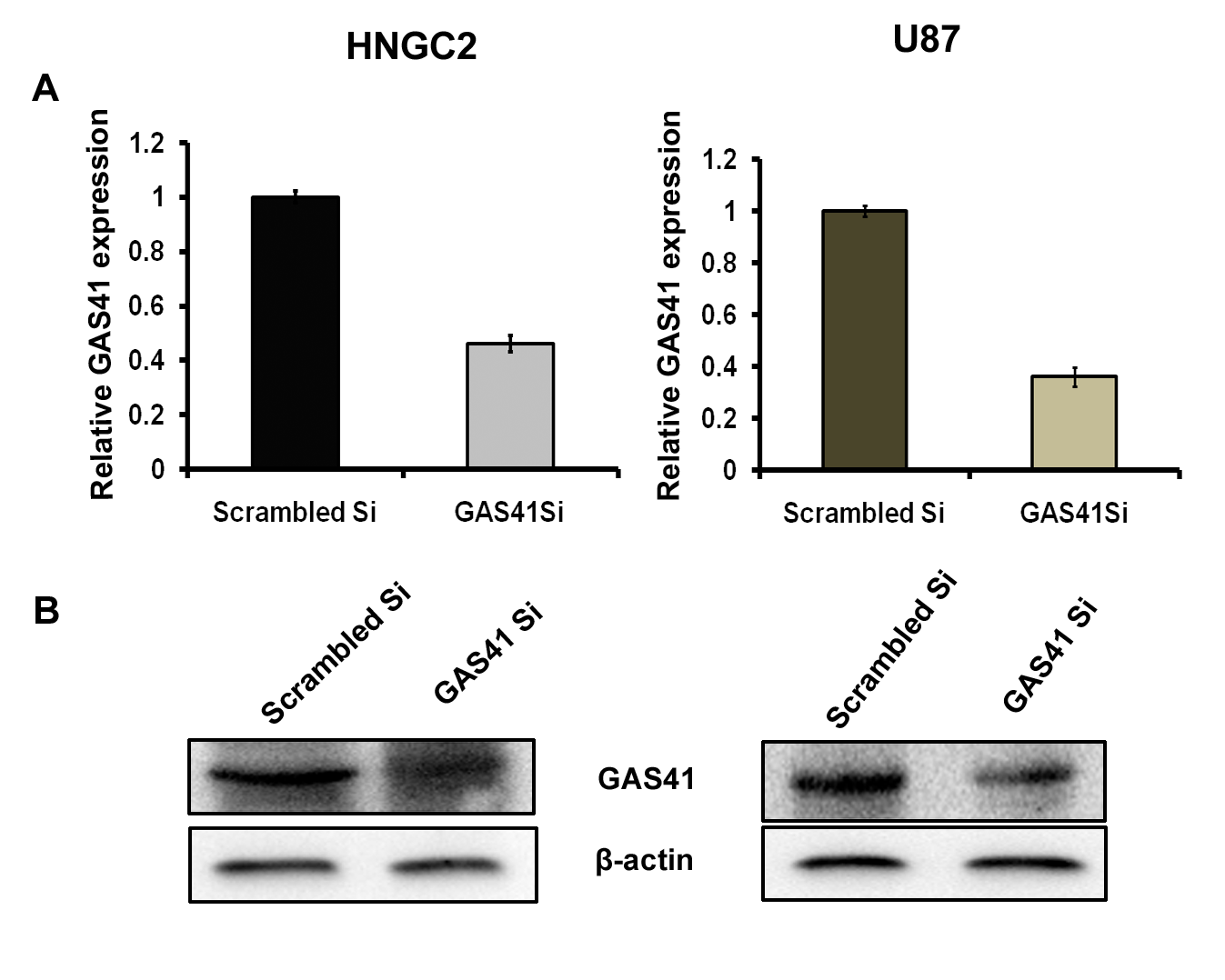

Supplement: S2 Fig — (A) qRT-PCR showing GAS41 mRNA expression in HNGC2 and U87 cell lines. Bars represent relative expression of GAS41 normalized to GAPDH. (B) Western blots showing GAS41 protein expression after cells were transfected with GAS41 specific siRNA. (TIF) [file pone.0159092.s002.tif]

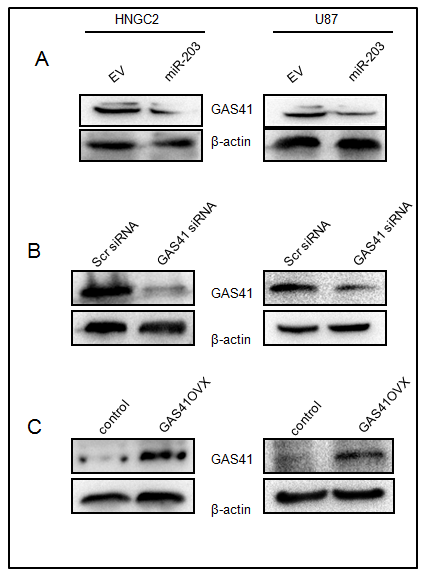

Supplement: S3 Fig — (A) Western blot analysis of GAS41 in HNGC2 and U87 cells transfected with Empty vector (EV) and miR-203 plasmid construct. (B) Western blot analysis showing the expression of GAS41 in HNGC2 and U87 after cells was introduced with scrambled siRNA (scr siRNA) and GAS41 siRNA (C) Western blot showing the over expression of GAS41 in HNGc2 and U87 after cells were transiently transfected with only vector and pCMV-Tag1-GAS41 over expressing construct. For all β-actin serve as loading control. (TIF) [file pone.0159092.s003.tif]

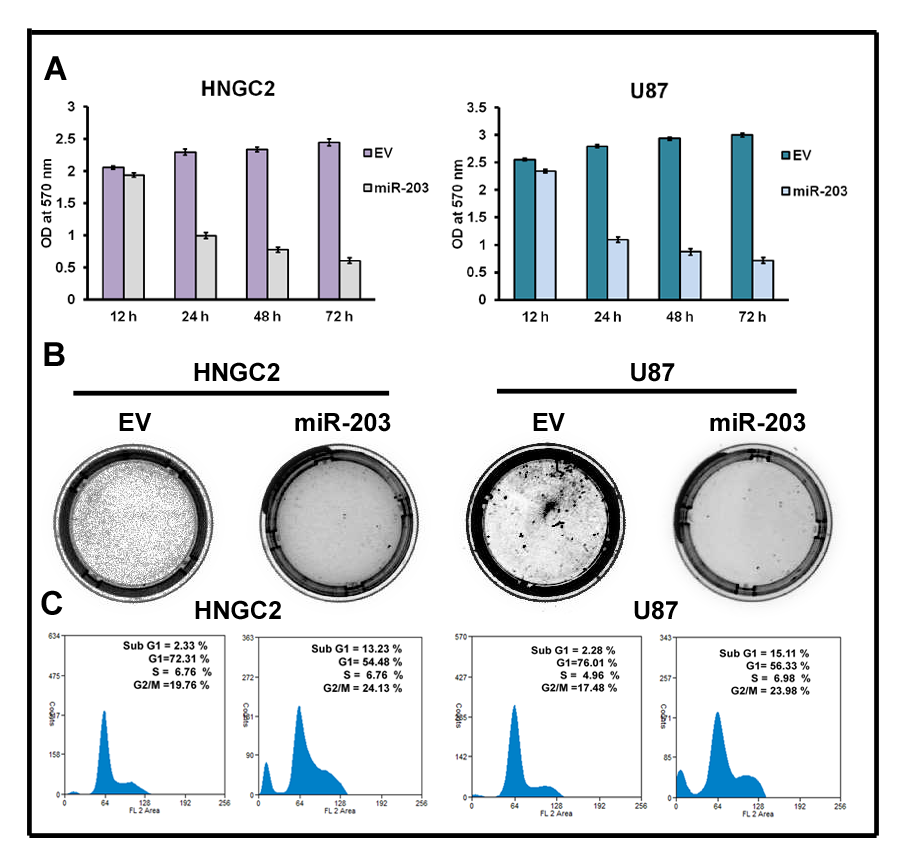

Supplement: S4 Fig — miR-203 inhibit glioma proliferation and induce apoptosis (A) Cell viability studies in HNGC2 and U87 cells after transfection either with empty vector or miR-203 over-expressing vector at different time intervals (12,24,48 and 72h) (B) Colony formation assay after miR-203 transfection into HNGC2 and U87 cells. (C) Flow cytometry analysis of cell cycle demonstrating apoptosis in HNGC2 and U87 cells after transfection with miR-203 or empty vector. (TIF) [file pone.0159092.s004.tif]
