# Supplementary material for: Regulation of Cell Proliferation and Migration by miR-203 via GAS41/miR-10b Axis in Human Glioblastoma Cells
Source: PLoS One. 2016 Jul 28;11(7):e0159092. doi: 10.1371/journal.pone.0159092 (PMC4965126; doi:10.1371/journal.pone.0159092)
Supplement: S1 Table — (DOCX) [file pone.0159092.s005.docx]

**Supplementary Table 1: primer list**

| **Primer Name** | | **Primer Sequence (5’-3’)** |
| --- | --- | --- |
| GAS-41 CDS forward primer | | AAAAAGCGGCCGCATGTTCAAGAGAATGGCCG |
| GAS-41 CDS reverse primer | | CGCGGATCCTTATATGTCTTTTGCTTGGTCATC |
| GAS-41 UTR forward primer | | CCGCTCGAGCCCACAGTTCTCATGAGAACTTGGTAGTAAGC |
| GAS-41 UTR reverse primer | | CGCGCGCGGCCGCTAACTGTATTGAGGGAGTTCCAGAAAG |
| GAS-41-RT forward primer | | TAAAGGGTGTTACTATCGTTAAACCAA |
| GAS-41-RT reverse primer | | TCTTAAAGGATTGCCATAGCTTTC |
| Pre-miR-203 forward primer | | CCGCTCGAGCTCGTCTAAGGCGTCCGGTACG |
| Pre-miR-203 reverse primer | | CCGGAATTCCGGAGTTTCGAGGTGCGG |
| miR-203-SDM forward primer | | GGCATCAAAATGTCATTATAAAGTATTACTTGTACAAAT |
| miR-203-SDM reverse primer | | ACTTTATAATGACATTTTGATGCCTGTCAAAAGGGTAAT |
| hsa- miR-203 forward primer | | GTGAAATGTTTAGGACCACTAG |
| hsa- miR-10b forward primer | | TACCCTGTAGAACCGAATTTGTG |
| U6 snRNA | | CGCAAGGATGACACGCAAATTC |
| P21 FL forward primer | | CAGAGGTACCCTTCCTAGGCAGCTTCTGCAGCC |
| P21 FL reverse primer | | CAG AAAGCTTGTATATCAGGGCCGCGCTGAGC |
| P21 -2500 to -1400 forward primer | | CAGAGGTACCCTTCCTAGGCAGCTTCTGCA |
| P21 -2500 to -1400 reverse primer | | CAG AAAGCTTGGAGGACAGGCTTCTTTCTCC |
| P21 -1400 to +100 forward primer | | CAGAGGTACCCCGAGGTCAGCTGCGTTAGA |
| P21 -1400 to +100 reverse primer | | CAGAAAGCTTCGCTCTCTCACCTCCTCTGA |
| Pri-miR-203 forward primer | | TCGGGGGCTCCTCTCTCC |
| Pri-miR-203 reverse primer | | CCCCTGACTGTGACTCTG |
| P53si RNA sense | CAGUCUCCCUCCCGCCAUAUU | |
| P53siRNA anti sense | AAUAUGGCGGGAGGUAGACUG | |
| Anti-miR-203 | TAGTGGTCCTAAACATTTCA | |
| GAS41 siRNA | GAGCUUAAGGAGAGAUUAAdTd | |
| P53 FOR | GTTCCGAGAGCTGAATGAGG | |
| P53 REV | TCTGAGTCAGGCCCTTCTGT | |
| GAPDH FOR | CTCATGACCACAGTCCATGC | |
| GAPDH REV | TTCAGCTCTGGGATGACCTT | |
